# Protection by the *NDI1* Gene against Neurodegeneration in a Rotenone Rat Model of Parkinson's Disease

**DOI:** 10.1371/journal.pone.0001433

**Published:** 2008-01-16

**Authors:** Mathieu Marella, Byoung Boo Seo, Eiko Nakamaru-Ogiso, J. Timothy Greenamyre, Akemi Matsuno-Yagi, Takao Yagi

**Affiliations:** 1 Division of Biochemistry, Department of Molecular and Experimental Medicine, The Scripps Research Institute, La Jolla, California, United States of America; 2 Pittsburgh Institute for Neurodegenerative Diseases and the Department of Neurology, University of Pittsburgh, Pittsburgh, Pennsylvania, United States of America; University of Auckland, New Zealand

## Abstract

It is widely recognized that mitochondrial dysfunction, most notably defects in the NADH-quinone oxidoreductase (complex I), is closely related to the etiology of sporadic Parkinson's disease (PD). In fact, rotenone, a complex I inhibitor, has been used for establishing PD models both *in vitro* and *in vivo*. A rat model with chronic rotenone exposure seems to reproduce pathophysiological conditions of PD more closely than acute mouse models as manifested by neuronal cell death in the substantia nigra and Lewy body-like cytosolic aggregations. Using the rotenone rat model, we investigated the protective effects of alternative NADH dehydrogenase (Ndi1) which we previously demonstrated to act as a replacement for complex I both *in vitro* and *in vivo*. A single, unilateral injection of recombinant adeno-associated virus carrying the *NDI1* gene into the vicinity of the substantia nigra resulted in expression of the Ndi1 protein in the entire substantia nigra of that side. It was clear that the introduction of the Ndi1 protein in the substantia nigra rendered resistance to the deleterious effects caused by rotenone exposure as assessed by the levels of tyrosine hydroxylase and dopamine. The presence of the Ndi1 protein also prevented cell death and oxidative damage to DNA in dopaminergic neurons observed in rotenone-treated rats. Unilateral protection also led to uni-directional rotation of the rotenone-exposed rats in the behavioral test. The present study shows, for the first time, the powerful neuroprotective effect offered by the Ndi1 enzyme in a rotenone rat model of PD.

## Introduction

Parkinson's disease (PD) is one of the most common neurodegenerative disorders affecting people over 60 years old [1]. The most notable pathological aspect of PD is the loss of the nigrostriatal pathway and, more precisely, of the dopaminergic neurons in the substantia nigra (SN). It should be noted that more than 95% of PD is sporadic but not autosomal. Attempts have been made to develop therapies for a number of targets such as monoamine oxidase B, dopamine level, growth factors, oxidative stress and apoptosis. Unfortunately, these agents are known to be only moderately effective for PD [2]–[5]. Therefore, it is important to develop new therapies for PD on the basis of innovative concepts.

Recently, it has been reported that sporadic PD is associated with dysfunction of mitochondria, particularly that of the proton-translocating NADH-quinone oxidoreductase (complex I) [6], [7]. In fact, it is known that many patients of PD have decreased complex I activity [7], [8]. Furthermore, complex I inhibitors such as 1-methyl-4-phenyl-1,2,3,6-tetrahydropyridine (MPTP), rotenone and acetogenins have been demonstrated to induce Parkinsonian symptoms in animals [9]–[11].

The alternative NADH-quinone oxidoreductase of *Saccharomyces cerevisiae* mitochondria (Ndi1) is composed of a single polypeptide which is encoded by the *NDI1* gene, and is insensitive to complex I inhibitors [12], [13]. Therefore, we advocated that yeast Ndi1 protein may be useful to protect against the symptoms of PD. So far the Ndi1 protein can be functionally expressed in rodent systems both *in vitro* and *in vivo* [14]–[16]. In addition, we demonstrated that Ndi1 protein has protective effects against inhibition of NADH oxidase activity by complex I inhibitors and overproduction of ROS caused by complex I defects in vitro [17]. We showed that the Ndi1 protein protected against neurodegeneration and behavioral deficits of acute MPTP mouse model of PD [18], [19]. More recently, rotenone rat models of PD have been developed that reproduce many biochemical and behavioral characteristics observed in human PD such as irreversible cell death of dopamine neurons and formation of Lewy body-like cytoplasmic inclusions [20]–[22]. In order to evaluate the Ndi1 protein as a potential therapeutic agent for PD, it is a prerequisite to investigate whether the Ndi1 protein is capable of protecting the nigrostriatal system against Parkinsonian symptoms in rotenone rat models of PD. In this paper, we present the results in support of great potential of the Ndi1 protein as an agent to retard PD.

### Experimental Procedure

#### Animals and experimental design

Forty two 5-month-old male Sprague-Dawley rats were used for this study. All animals were given standard rat chow and water ad-libitum. The rat's weight was approximately 480–520 g at the time of surgery. Animals were first divided in 2 groups: one group received unilateral intracerebral injection of recombinant adeno-associated virus (AAV) harboring the *NDI1* gene (serotype 5; Applied Viromics) and the other group received intracerebral PBS injection. Then, each group was further divided into 2 groups: one group received subcutaneous injection of rotenone microspheres and the other received subcutaneous injection of the same amount of PBS microspheres. The four groups were labeled as “NDI1” (*NDI1* injection into the right SN and subcutaneous injection of PBS, this group was composed of 5 animals), “NDI1+rotenone” (*NDI1* injection into the right SN and subcutaneous injection of rotenone, this group was composed of 20 animals), “control” (PBS injection into the right SN and subcutaneous injection of PBS, this group was composed of 3 animals) and “control+rotenone” (PBS injection into the right SN and subcutaneous injection of rotenone, this group was composed of 14 animals). The quantity of microspheres injected was adjusted in such a way that each rat receives 100 mg of rotenone per kg of body weight. The microspheres were assumed to incorporate 100% of rotenone used for the preparation. Subcutaneous injections of microspheres were performed 45 days after virus injections to allow a complete expression of the Ndi1 protein in the brain. Thirty days after the microsphere injection, the first round of behavioral tests were carried out and brain samples were collected from some of the animals. Half of the samples were used for immunohistochemistry and the other half for chemical determination of dopamine and its metabolites. After another 30 days the remaining animals were tested exactly in the same way.

#### Surgery

Three-µl of AAV serotype 5 carrying the *NDI1* gene (3.1×10^12^ genome copy/ml) or 3 µl of PBS were injected intra-cerebrally at 240 nl/min into the SN of the rats as previously described [16]. The injection target had the following stereotaxic coordinates: antero-posterior from the bregma, -5.3 mm; media-lateral from the bregma, -2.4 mm; dorso-ventral from the dural surface, 7 mm.

#### Preparation of microspheres

Biodegradable polymer of poly(DL-lactide-co-glycolide) (PLGA; lactide∶glycolide 75∶25, mol. wt. 90,000–126,000, Sigma) containing rotenone were prepared according to an emulsion solvent evaporation/extraction method. Rotenone (75 mg) and PLGA (400 mg) were dissolved in 15 ml of dichloromethane. The solution was vortexed for at least 15 min at ambient temperature. The organic phase was poured into ice-cold 4% (w/v) polyvinyl alcohol (hot water soluble; Sigma). The emulsion was stirred at maximum speed for 1 hour in hermetic condition. Then the seal was broken in order to evaporate the dichloromethane for 4 hours at ambient temperature. The microspheres were collected by centrifugation, washed with distilled water, dried, and kept at −20°C until use. For controls, rotenone was replaced with PBS. The average diameter of the beads was estimated to be 50 µm.

#### Histological analysis

Brain samples were collected after perfusion with 4% paraformaldehyde (v/w, pH 7.4) and were placed in 4% paraformaldehyde for 2 hours at 4°C and in 20% sucrose (v/w, pH 7.4) overnight. Coronal sections of 30 µm were collected onto superfrost slide (Fisher) and stored at −20°C. 7,8-dihydro-8-oxo-deoxyguanine (8-oxo-dG) staining was performed as previously described [23]. Briefly, brain slices were incubated in 70% ethanol precooled to −20°C for 10 minutes on ice. After rinsing with PBS the slides were soaked in 37°C PBS supplemented with 100 µg/mL RNase A, DNase-free for 1 hr. Staining was enhanced by incubation of the slices with 4 N HCl for 7 minutes followed by neutralization with two 2-minutes washes with 500 mM Tris-HCl, pH 7.4. Blocking for immunostaining was done in TBS containing 5% FBS, 5% horse serum and 0.05% Triton-X-100 for 2 hr. The slides were incubated with primary mouse anti-8-oxo-dG antibody (1/300) in TBS containing 2.5% FBS, 2.5% horse serum and 0.05% Triton-X-100 overnight at 4°C and then with FITC-labeled anti-mouse secondary antibody (1/100) in the same medium for 5 hr at room temperature. Alpha-synuclein and ubiquitin staining were performed with specific antibodies diluted at 1/300 in PBS, 10% horse serum and 0.1% triton X-100. Both antibodies were revealed with a biotinylated secondary antibody and the ABC Elite kit (Vector Laboratories). For the Ndi1 protein and tyrosine hydroxylase (TH) revelation using fluorescence, brain slices were boiled twice for 5 min in 10 mM Tris-Cl, pH 10, washed with PBS, and blocked using Image-iT FX (Invitrogen) for 3 hrs. Sections were then incubated overnight at 4°C with primary antibody in TBS, 10% horse serum, 0.1% triton X-100 (Ndi1: 1/300, TH: 1/300; Calbiochem). The anti-Ndi1 protein antibody was revealed with a specific horseradish peroxidase conjugated goat-anti-rabbit IgG (1/1000; Calbiochem) and the TSA Plus Fluorescein system kit (PerkinElmer). The TH antibody was revealed with Alexa fluor 568 goat anti mouse IgG (Invitrogen). The level of TH in the striatum was estimated by using DAB (3,3′ Diaminobenzidine; Sigma) staining. The specific TH antibody was revealed with biotinylated secondary antibody and the ABC Elite kit (Vector Laboratories). For the evaluation of NADH dehydrogenase activity, brains were snap frozen and sliced into 30 µm sections. The sections were then stained for 5 min at 37°C with a solution containing 2 mg/ml nitro blue tetrazolium, 0.5 mM NADH in 50 mM Tris-Cl, pH 7.6. The slices were washed twice with TBS and destained with increasing concentrations of acetone solutions (30%, 60%, 90%). The slices were dried and mounted with Permount (Fisher).

#### Chemical analysis

For the detection of rotenone in plasma, 200 µl of blood was harvested from the tail vein every week and the plasma was kept frozen at −80°C until use. Rotenone was extracted from the plasma with 1 volume of dichloromethane (Sigma) for 15 minutes. After centrifugation the organic phase was evaporated by Speedvac (Savant) for 5 min. These steps were repeated twice. The pellet was dissolved in 30 µl of acetonitrile and samples of 15 µl were analyzed on a C_18_ column (Waters Spherisorb 5 µm ODS2, 4.5×150mm) at 214 nm. The mobile phase was composed of 70% methanol in H_2_O and the flow rate was set at 1 ml/min. The retention time of the rotenone peak was around 10.5 min.

For the detection of dopamine and its metabolites, the brains were quickly collected, dissected and flash frozen on dry ice. The right and left striata were isolated and weighed. Each sample was homogenized in ice-cold 0.2 M perchloric acid (0.5ml of acid per 100 mg of wet tissue) using sonication. The homogenate was kept on ice for 30 min and centrifuged at 20,000 g, for 15 min at 4°C. The supernatant was analyzed on an Eicompak SC-30DS column (3×100 mm). The mobile phase was composed of 80 mM citrate-acetate buffer (pH 3.5) containing 176 mg/l sodium octane sulfonate, 4 mg/l EDTA and 20% (v/v) methanol. The signal was detected through an electrochemical detector (EPC-500) set at +750 mv (Eicom) coupled to a Power Chrom data processor (Eicom). The flow rate was set at 0.4 ml/min. The amount of dopamine at day 30 or day 60 didn't show significant difference and, therefore, the values were compiled into one histogram.

#### Behavioral testing

The rotational behavior of rats was measured by placing the animal into a round shape basket of 48 cm diameter over which a camcorder was mounted. The rats were placed in the basket 15 minutes before they were injected subcutaneously with 1.5 mg/kg apomorphine hydrochloride (Sigma, Saint Louis, MO). The number of clockwise and counter-clockwise turns and the speed of each animal were monitored for 30 min, and the percentage of completely lateralized animals per group was evaluated.

## Results

### Evaluation of the rotenone rat model

**Figure 1:**
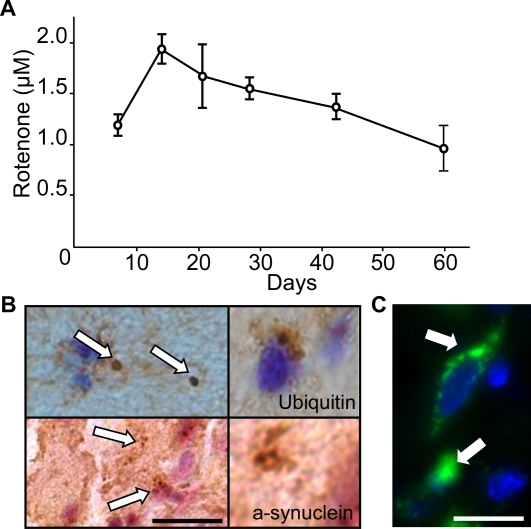
Characterization of the rotenone rat model. Rats received a subcutaneous injection of microspheres encapsulating rotenone. A: Concentration of rotenone in the plasma was determined on an HPLC system from 200 µl of blood samples drawn from the tail vein from 4 rats every week for 60 days. Values are given with standard deviation (SD). B: Rat brain coronal sections at the level of SN were subjected to immunohistochemical staining with antibody against α-synuclein or ubiquitin. Left panel, Lewy body-like cytoplasmic inclusions in SN neurons (arrows). Scale bar is 10 µm. Right panels, images at a higher magnification. C: The rat SN sections were stained with antibody specific for 8-oxo-dG. Oxidatively modified DNA appeared in green. Nuclei were stained in blue with DAPI. The image shows the damage to both mitochondrial DNA (left arrow) and nuclear DNA (right arrow) in SN neurons. Scale bar is 15 µm.

A few methods are available to administer rotenone in rats. For example, intravenous infusion via minipump [20] or intraperitoneal injection [24] may be used. Our preliminary experiments conducted with an intraperitoneal injection of rotenone, emulsified in oil, turned out to exert noxious effects; the rats presented liver necrosis and large weight loss (unpublished data). Subcutaneous delivery of rotenone, on the other hand, was reported to be more efficient in altering selectively the dopaminergic cells [22]. To eliminate toxic effects derived from the materials used for injections such as organic solvents, we carried out subcutaneous injections of biodegradable microspheres containing rotenone, a method originally adopted by Huang et al. [25]. After the injection, we monitored the concentration of rotenone in the plasma. As shown in Figure 1A, there was an initial increase in the rotenone concentration during the first 2 weeks which was followed by a gradual decrease. The rotenone concentration remained above 1 µM throughout the period of our experiments (60 days) presumably due to a slow and constant release of rotenone from the polymer undergoing disintegration in the animal body [26]. The rats lost about 2% of their body weight during the first 15 days after the injection which coincided with an increase in the rotenone concentration in the plasma, but there was no further loss throughout the rest of the period (data not shown). After 30 or 60 days of rotenone exposure, we examined the brain for the extent and nature of the damage to the nigral neurons. One of the features of PD is formation of filamentous amyloid deposits, Lewy bodies. Such cytoplasmic aggregations were observed in rotenone rat models of PD [22], [27]. We performed immunohistochemical staining of brain sections using an antibody against α-synuclein or ubiquitin and detected Lewy-body like inclusions in SN neurons of all the rats that were exposed to rotenone (Figure 1B). Furthermore, Nissl staining of the brain sections revealed a substantial decrease in the number of viable neurons in the SN of rotenone-treated animals (see below). The SN neurons of the rotenone-treated rats also exhibited extensive staining with antibody against 8-oxo-dG, indicating oxidative modification of DNA (Figure 1C). The oxidative damage was observed in both mitochondrial and nuclear DNA.

### Unilateral delivery of the *NDI1* gene to the rat SN and the effects of rotenone treatment

**Figure 2:**
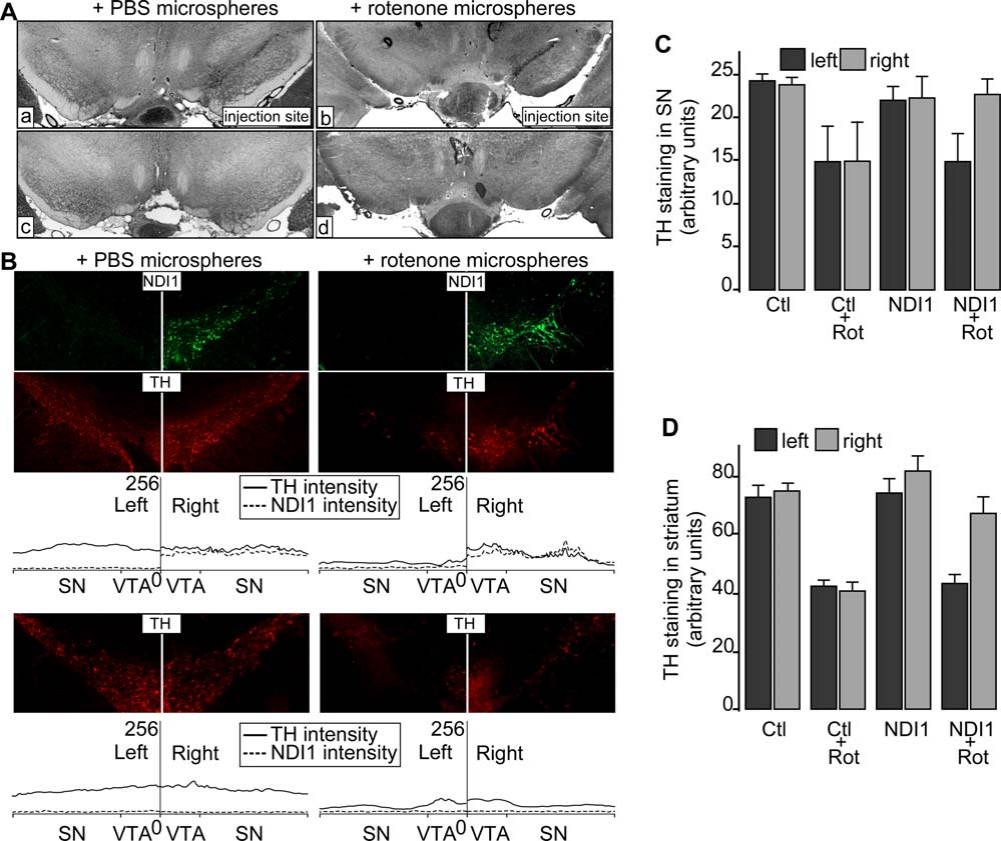
Expression of the Ndi1 protein in the rat SN and its effect on the TH levels in the SN and striatum after rotenone exposure. Rats received a unilateral injection of recombinant AAV carrying the *NDI1* gene targeted at the right SN. Control rats were injected with PBS. After the expression of the Ndi1 protein has been established, microspheres containing rotenone were injected subcutaneously. For control, microspheres with PBS were used. Thirty or 60 days later, brains were collected for analysis. A: After 30 days of rotenone exposure, brain sections at the level of SN were stained for the NADH dehydrogenase (diaphorase) activity. The right SN that received the *NDI1* gene showed high NADH dehydrogenase as revealed by dense staining in both rotenone-treated (b) and PBS-treated (a) group. Non-NDI1 control rats did not exhibit discernible staining (c and d). B: Brain sections from the same groups of rats as A were double-stained for TH (red) and the Ndi1 protein (green). The staining intensities across the right SN (*NDI1* injection side) and the left SN are plotted in arbitrary units below each set of the images. Each picture displays a representative section of an animal from each group. C: Rats expressing the Ndi1 protein (NDI1) and non-NDI1 control group (Ctl) were treated with rotenone (Rot) for 60 days and immunohistochemical staining for TH staining were carried out as in B. The fluorescence images of the brain sections were collected and the staining intensity of TH from the left and the right SN was statistically analyzed (student T-test) using ImageJ [39]. Note that the right SN received the *NDI1* injection. The number of sections used was; Ctl (n = 6), Ctl+Rot (n = 9), NDI1 (n = 18) and NDI1+Rot (n = 18). Images in each group were collected from evenly spaced sections that cover the entire SN in which the presence of the Ndi1 protein was detected. Error bars represent standard deviation. D: Levels of TH staining in the left and right striatum from the same four groups of rats as in C are compared. The right striatum is ipsilateral to the SN expressing the Ndi1 protein. Representative images of DAB staining of TH are given in Figure S2. The number of sections used for analysis was; Ctl (n = 6), Ctl+Rot (n = 9), NDI1 (n = 9) and NDI1+Rot (n = 18). Error bars represent standard deviation.

Using the rotenone rat model of PD, we carried out the experiments that allow evaluation of capability and efficiency of the Ndi1 protein as a therapeutic agent. We have previously reported that the Ndi1 protein was functionally expressed in the rat SN following a stereotaxic injection of recombinant AAV carrying the *NDI1* gene [16]. However, the level of the Ndi1 protein expression was variable and, therefore, not most suitable for our aims. In an attempt to achieve a consistently high level and a wider area of the Ndi1 protein expression in the SN, we selected AAV serotype 5 [28] which was reported to deliver the transgene in the SN more efficiently than AAV serotype 2 that was used in our previous experiments [16]. The injection point was set at 0.4 mm above the SN in order to avoid any physical damage to the dopaminergic pathway during the surgery. In all animals tested, a single, unilateral injection of the viral particles resulted in the expression of the Ndi1 protein in the entire SN spanning 1.8 mm sagittally. The wide distribution of the Ndi1 protein in the SN dopaminergic neurons was evident in a coronal section of the brain double-stained with antibodies to the Ndi1 protein and TH (Figure S1, A and B). It was also confirmed that the Ndi1 protein was localized to mitochondria (Figure S1, C–E) just as we reported earlier using cultured cells of various origins [14], [15], [29] as well as in rodents [16], [18]. Near perfect colocalization of the Ndi1 protein and mitochondria was most clearly visualized in a 3D image of an SN neuron constructed from a series of confocal images of a brain section double-stained for the Ndi1 protein and the α-subunit of F_1_-ATPase (Movie S1). The level of TH and the amount of dopamine were not affected by the Ndi1 protein expression. The expressed Ndi1 protein was enzymatically active as seen in dense staining derived from NADH dehydrogenase activity in the SN on the injected side (Figure 2A).

We subjected the rats unilaterally expressing the Ndi1 protein to rotenone exposure. The NADH dehydrogenase activity derived from the Ndi1 protein was not affected by rotenone in agreement with insensitivity of the enzyme to this chemical (Figure 2A). Rotenone intoxication caused approximately 45% decrease in the TH level of the SN that did not receive the *NDI1* gene but almost no effect on the SN expressing the Ndi1 protein (Figure 2B and 2C). In fact, there was a good correlation between the presence of the Ndi1 protein and the TH staining as can be seen in Figure 2B in which profiles of the staining intensity across the SN region are compared. It should also be noted that the presence of the Ndi1 protein did not cause any change in the TH level in the SN. The SN projects to all areas of the striatum. In PD, severe neurodegeneration of the striatal innervation derived from the SN neurons is observed. Therefore, we examined the level of TH in the striatum by histochemical staining of coronal brain sections (Figure S2). Statistical analysis of the images indicated that rotenone exposure led to approximately 50% decrease of TH on the contralateral striatum and that no discernible effect was observed on the ipsilateral striatum as expected (Figure 2D) in good agreement with the loss of TH in the SN.

**Figure 3:**
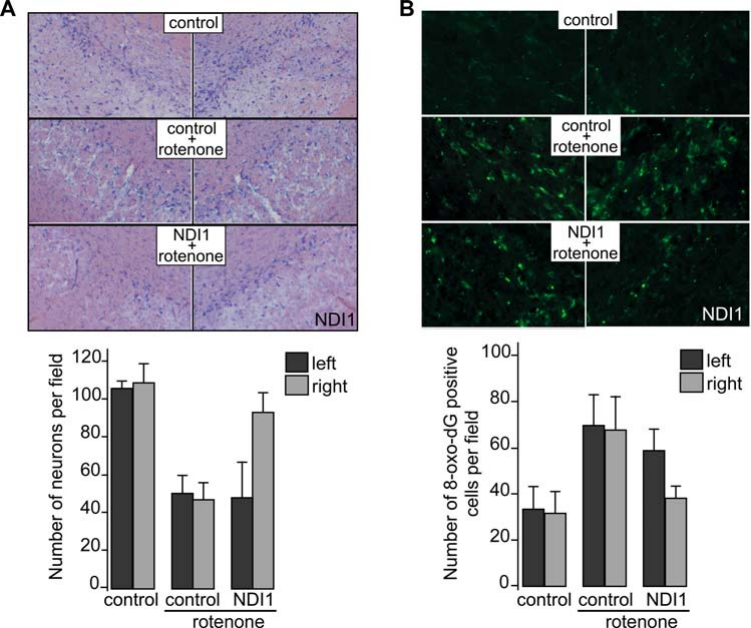
Prevention of rotenone-induced neuronal cell death and oxidative damage by expression of the Ndi1 protein. Rats were treated with the *NDI1* gene and rotenone and brain sections were prepared and processed for immunohistochemical staining as described in Figure 2. A: Sections were subjected to Nissl staining and representative images are displayed (upper panel). The number of viable neurons was compared between the left and right SN by counting Nissl-positive cells in a given area of the SN (lower panel). The number of sections used for analysis was; control (2 animals, 3 sections), control+rotenone (5 animals, 12 sections) and NDI1+rotenone (9 animals, 9 sections). In each group, images were collected from the sections with the matching anterior-posterior position and, when multiple sections were used in a given animal, they were separated by at least 30 µm to eliminate possible span of neuron cell bodies over multiple sections. B: Sections were stained for 8-oxo-dG to evaluate oxidative damage to DNA (upper panel). The SN neurons that are positively stained were counted (lower panel). The number of sections used for analysis was; control (2 animals, 3 sections), control+rotenone (3 animals, 5 sections) and NDI1+rotenone (3 animals, 6 sections). Selection of the sections was done as described in A. Statistic analysis was done using student T-test. Error bars represent standard deviation.

As described above, rotenone exposure resulted in the appearance of cytoplasmic aggregations. This Lewy-body like structure seems to form less frequently, on the ipsilateral side, in the group of rats that were injected with the *NDI1* gene. However, the total number of such inclusions was too small to obtain statistically reliable data. Neuronal cell death is another key observation in the rotenone rat model of PD. Approximately 50% of the SN neurons died in our rotenone model (Figure 3A). Comparison of the number of SN neurons between the injection side and the opposite side clearly showed that the Ndi1 protein expression prevented neuronal cell death in rotenone-treated animals. The preferential death of the nigrostriatal neurons under rotenone exposure may result from the synergic effect of ROS production by complex I inhibition and the presence of dopamine by way of production of oxyradicals and reactive intermediates [30], [31]. Our earlier experiments using rat PC12 cells demonstrated that the Ndi1 protein impedes the production of ROS triggered by complex I inhibition presumably by taking over electron transfer activity from endogenous complex I [17], [29], [32]. To examine whether the Ndi1 protein can exert the same ROS-reducing effect *in vivo*, we assessed the extent of oxidative DNA modifications in the SN neurons. Again, the data from the Ndi1-expressing SN exhibited almost no effect by rotenone whereas the opposite side underwent the same degree of damage as non-Ndi1 controls (Figure 3B).

### Dopamine levels in the striatum after rotenone exposure and effect of expressing the Ndi1 protein

**Figure 4:**
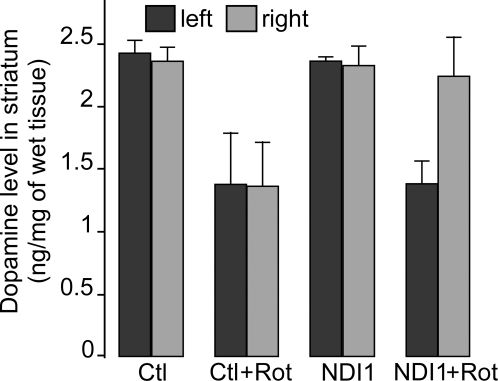
Reduction of striatal dopamine by rotenone exposure and its retention by expression of the Ndi1 protein. Procedures for the *NDI1* gene injection and rotenone exposure were the same as those described in Figure 2. The striatal portions of unfixed brains were dissected and homogenized. Samples were analyzed for dopamine contents on an HPLC system coupled with an electrochemical detector. The dopamine contents were determined for the left and right hemisphere from the four groups, Ctl (n = 3), Ctl+Rot (n = 8), NDI1 (n = 3) and NDI1+Rot (n = 11). n = number of animals. All values are presented with standard deviation.

A decrease in dopamine content is associated with PD symptoms and measurement of dopamine is commonly used for assessment of neurodegeneration. In our rat model, the dopamine level in the striatum of animals exposed to rotenone was decreased by 45% of the control group (Figure 4). In the group that received the *NDI1* gene in the SN unilaterally, the striatum contralateral to the SN of the injection side lost dopamine to about the same extent as the rotenone group without the *NDI1* gene. The ipsilateral striatum, however, retained the same concentration of dopamine as the non-rotenone group. These results demonstrate that the Ndi1 protein expression in the SN protected the nigrostriatal pathway in the rotenone-administered rats.

### Evaluation of the behavior of rats

**Figure 5:**
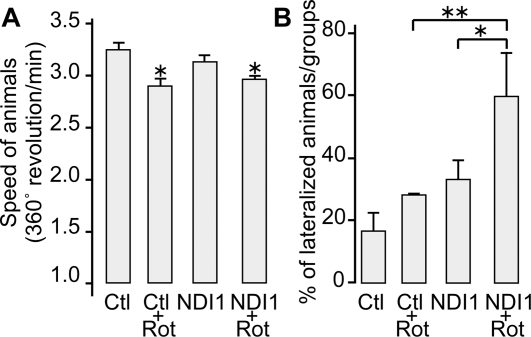
Behavioral tests of rotenone-exposed rats. Rotenone exposure was carried out as described in Figure 2. Each rat was placed in a round bucket after apomorphine administration and the movement was video-taped for 30 min. Data were analyzed for the four groups; Ctl (n = 3), Ctl+Rot (n = 14), NDI1 (n = 5) and NDI1+Rot (n = 20). n = number of animals. A: Speed of movement of rats was calculated as the number of full turns per minute. B: The number of rats exhibiting fully lateralized turning in either direction was counted. Histograms represent percentage of animals turning only in one direction. (*: p<0.05; **: p<0.01). All values are presented with standard deviation.

In our rotenone model, the rats were seemingly active in movement throughout the period of the experiments. We measured the speed of movement of each animal by counting the number of full turns per minute after an apomorphine injection. The rotenone treatment slowed down the animals only slightly but with a statistically significant difference (Figure 5A). A similar slowdown was observed in the animals expressing the Ndi1 protein. It should be noted that we have deliberately chosen a unilateral delivery of the *NDI1* gene to achieve unequivocal assessment of the consequences of the target gene expression by comparing the two sides in the same animal. The impact on the overall movement may be rather insignificant. However, this unilateral treatment made it possible to carry out behavioral tests involving turning preferences. A difference in the integrity between the right and the left side of the dopaminergic pathways should lead to an asymmetric behavior. Individual animals placed in a container were monitored for their lateralization after an apomorphine injection (Movie S2). Because the direction of rotation induced by apomorphine seems to be dependent on multiple factors, we decided to count the number of animals exhibiting 100% lateralized rotation irrespective of the direction (for review see [33]). As illustrated in Figure 5B, in the groups of rats that were not exposed to rotenone, a small difference was noticed between the NDI1 group and the non-NDI1 control group. However, this difference was not statistically significant (p = 0.242, student T-test). In the animals bearing the Ndi1 protein in the SN, rotenone boosted the lateralized turning significantly. The preferential turning of the NDI1 group of rats completely agrees with the histochemical and biochemical analyses described above.

## Discussion

The concept of complementing mammalian complex I with the alternative NADH dehydrogenase was first tested in a variety of cultured cells in our group [15], [29], [34] and, more recently, in other laboratories [35], [36]. The cell lines employed ranged from normal cells of different origins such as kidney cells, fibroblasts and neurons to complex I-deficient cells derived from patients or cybrid cells. In all cases, the results were positive and the Ndi1 protein seems to be fully functional as a member of the host respiratory chain and can restore NADH oxidation to complex I-impaired cells. The first attempt to take this effort to the next step of demonstrating it in live animals was quite successful. The Ndi1 protein expressed in the SN of mouse brain was able to compensate for loss of complex I in dopaminergic neurons in MPTP-exposed mice [18]. The initial success prompted us to carry out the *in vivo* study using animals in a more detailed and well-defined manner. Advancement in this direction required improvement in at least two points.

First, we needed an efficient and reproducible method of expression of the Ndi1 protein in the rat brain. It is known that choice of the AAV serotype contributes to better expression of a therapeutic gene and more selective expression in terms of tissue specificity. Serotype 5 used in the present study turned out to be quite suitable for targeting dopaminergic neurons in the rat SN. The level and the spread of the Ndi1 protein in the SN remained unchanged for at least 5 months while our experiments were conducted. Second, we were able to establish a rat animal model of PD in which to carry out all the biochemical and behavioral experiments. The model follows in principle the chronic exposure of rotenone to cause PD-like neurodegeneration in combination with use of biodegradable polymers as the delivery tool of the chemical. The animals under our procedure suffered only a minimal loss of body weight throughout the period of the experiment and sustained strength to allow behavioral testing. Another point that should be noted regarding the current method is that we observe neuropathological deficiencies in >80% of the animals tested. This number is much higher than the previous model in which only 33–48% of the rats developed striatal lesions [20], [37]. A possible reason for the less variability of rotenone effectiveness in the current study may be that we used older (4 to 5 month-old) rats compared to the earlier studies (∼2 month-old). It should be pointed out that the data presented in this study came from all animals used without excluding any. Furthermore, the current rat model mimics human PD better than our own mouse model [18] in several aspects such as death of SN neurons and formation of Lewy body-like aggregations.

It is most obvious that the role of the Ndi1 protein as a replacement of malfunctioning complex I is to offer a shuttle from NADH to the downstream respiratory chain. However, there is an important consequence to securing an alternative route for NADH oxidation. That is prevention of overproduction of ROS by complex I. We reported earlier that, in mitochondria isolated from PC12 cells expressing the Ndi1 protein, ROS generation from complex I was significantly less compared to that of mitochondria from control cells [17]. This phenomenon is explained by the observation that the NADH concentration of the mitochondria carrying the Ndi1 protein remains low even when complex I is blocked [23] and that the resulting high redox potential in the matrix does not allow ROS formation by complex I [38]. It is well understood that ROS is a key factor in the mechanism of cell death in PD. We indeed demonstrated oxidative damage in the SN neurons in our rat model, and such damage was not observed in the Ndi1-expressing SN. It is conceivable that the Ndi1 protein *in vivo* is capable of keeping the NADH level low enough just as in cultured cells [23].

In addition to harmful ROS generation, cellular ATP levels might be involved in cell death caused by complex I defects. Whether this is the major event is still debatable because, for example, rotenone exposure does not seem to make any noticeable change in the ATP level [32]. Regardless, it has been reported that in cultured striatal neurons the Ndi1 protein sustains cellular ATP even in the presence of a complex I inhibitor [35]. Furthermore, we showed using PC12 cells that rotenone-induced loss of mitochondrial membrane potential is prevented by the presence of the Ndi1 protein [23].

The present study demonstrates, for the first time, that the yeast Ndi1 protein protects the nigrostriatal pathway in a chronic animal model of PD. Although the detailed mechanism of protection has yet to be fully understood, the successful action exerted by the Ndi1 protein in the animal model that closely mimics the pathophysiology of the disease is one critical step forward toward the goal of retarding PD by a novel strategy.

## Supporting Information

Figure S1 Expression of the Ndi1 protein in dopaminergic neurons of the substantia nigra (SN) of rat brain and its localization to mitochondria. Recombinant adeno-associated virus (serotype 5) carrying the *NDI1* gene was stereotaxically injected to the right SN. Coronal sections at the level of SN were subjected to immunohistochemical staining. (A, B) Double-staining of a section with antibody against Ndi1 (A) and tyrosine hydroxylase (TH) (B) showing the expressed Ndi1 throughout SN neurons. Scale bar is 500 μm. (C–E) Confocal microscopy images of a SN neuron double-stained with antibody to Ndi1 (C) and the α subunit of F1-ATPase (D). Localization of Ndi1 to mitochondria is clearly seen in the merged image (E). Scale bar is 8 μm.
(2.72 MB TIF)

Figure S2 Representative images of immunohistochemical staining of coronal sections at the level of striatum with antibody against TH. The staining intensity for TH from four groups of rats was statistically analyzed and the results were presented in Figure 2C.
(4.46 MB TIF)

Movie S1 3D images of a SN neuron double-stained with antibody against Ndi1 (green) and the α subunit of F1-ATPase (red). Immunohistochemical staining was carried out as described in Figures S1 C and D, and successive confocal images were collected along the Z-axis. 3D reconstruction of the cell was done for each color from the stacked images using OsiriX. The resulting movies were edited using Cinelerra to add a merged image and to rotate them in a synchronized manner.
(1.92 MB MOV)

Movie S2 Rotenone-treated rats were monitored for lateralized movement after apomorphine administration. Each rat was placed in a bucket and video-taped for 30 min. The first section of the movie is a non-NDI1 control rat and the second section is a rat that received the *NDI1* gene on the right SN. The movie is played at 4× acceleration.
(0.77 MB MOV)
