# Supplementary figures and images for: Protection by the NDI1 Gene against Neurodegeneration in a Rotenone Rat Model of Parkinson's Disease
Source: PLoS One. 2008 Jan 16;3(1):e1433. doi: 10.1371/journal.pone.0001433 (PMC2175531; doi:10.1371/journal.pone.0001433)

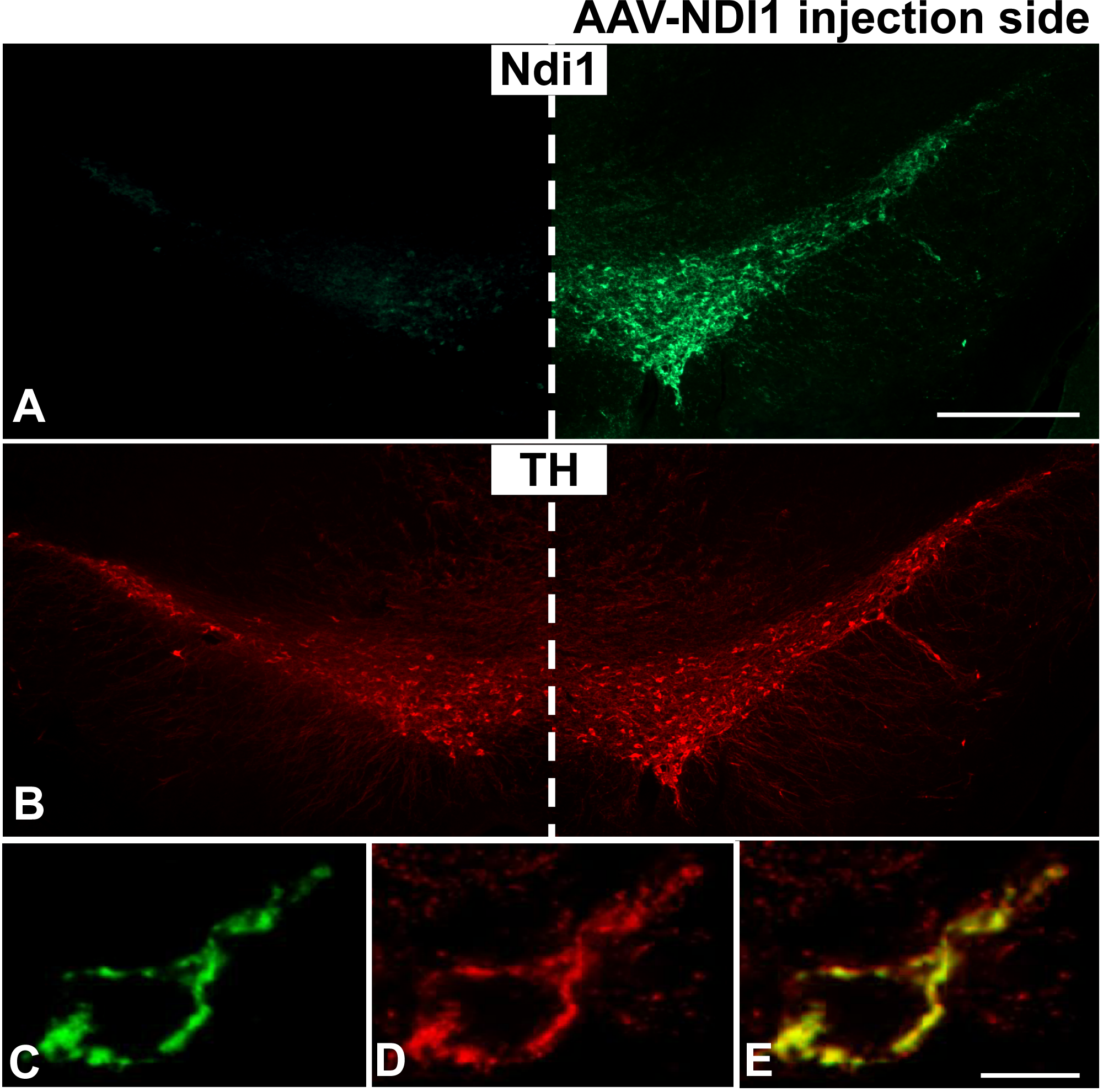

Supplement: Figure S1 — Expression of the Ndi1 protein in dopaminergic neurons of the substantia nigra (SN) of rat brain and its localization to mitochondria. Recombinant adeno-associated virus (serotype 5) carrying the NDI1 gene was stereotaxically injected to the right SN. Coronal sections at the level of SN were subjected to immunohistochemical staining. (A, B) Double-staining of a section with antibody against Ndi1 (A) and tyrosine hydroxylase (TH) (B) showing the expressed Ndi1 throughout SN neurons. Scale bar is 500 μm. (C–E) Confocal microscopy images of a SN neuron double-stained with antibody to Ndi1 (C) and the α subunit of F1-ATPase (D). Localization of Ndi1 to mitochondria is clearly seen in the merged image (E). Scale bar is 8 μm. (2.72 MB TIF) [file pone.0001433.s001.tif]

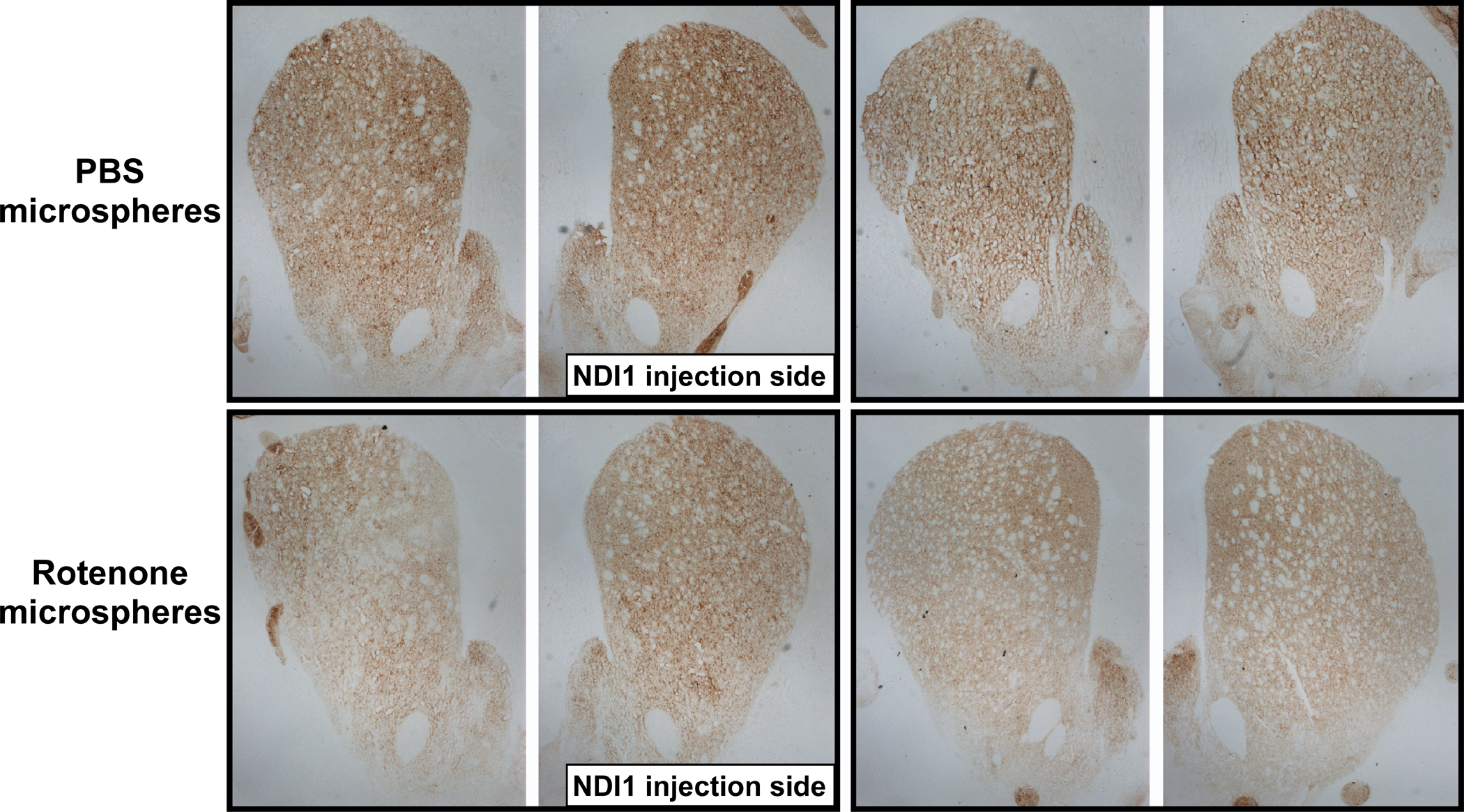

Supplement: Figure S2 — Representative images of immunohistochemical staining of coronal sections at the level of striatum with antibody against TH. The staining intensity for TH from four groups of rats was statistically analyzed and the results were presented in Figure 2C. (4.46 MB TIF) [file pone.0001433.s002.tif]
